# Genetic evolution, epidemic trends, and recombination dynamics of PRRSV-1 in China

**DOI:** 10.3389/fvets.2025.1632917

**Published:** 2025-08-05

**Authors:** Chengzhen Weng, Xinxin Huang, Zhian Chen, Minjia He, Beiwen Zhang, Hongxi Li, Jingrui Xie, Meichun Chen, Longxin Qiu, Xiaobing Li, Chong Cao, Hongbo Chen

**Affiliations:** ^1^Longyan College Life Science School, Longyan, China; ^2^Fujian Provincial Key Laboratory of Preventive Veterinary Medicine and Veterinary Biotechnology, Longyan, China; ^3^College of Animal Science, Fujian Agriculture and Forestry University, Fuzhou, China

**Keywords:** PRRSV-1, whole genome, amino acid site, genetic evolution, GP5, mutation

## Abstract

The persistent threat of porcine reproductive and respiratory syndrome virus (PRRSV) to the global swine industry is exacerbated by the virus’s high mutation rate and frequent recombination events. In China, the emergence of new PRRSV-1 strains in recent years has posed a significant challenge to the sustainability of pork production. This study systematically investigated the epidemiological patterns, genetic evolution, recombination dynamics, GP5 genetic diversity, and N-glycosylation variants of PRRSV-1 strains circulating in China. Whole-genome analysis demonstrated that Chinese PRRSV-1 isolates clustered within subtype 1, with BJEU06-1-like as the predominant subgroup and NMEU09-1-like as the secondary subgroup. Novel subgroups (new subgroups 1, 2, and 3), a new strain, GD2022, and an independent branch represented by strain GXFS20220129 were concurrently identified. High genetic diversity existed both within and between subgroups of Chinese PRRSV-1 strains. Whole-genome recombination has predominantly occurred through inter-subgroup exchange, primarily involving the BJEU06-1-like and Amervac-like lineages. Additionally, recombination events were identified between the field strain NVDC-FJ and the vaccine strain PRRSV1-CN-FJFQ-1-2023. Interestingly, the diversity of the ORF5 gene was consistent with that of the whole genome; however, there is a deviation in the phylogenetic tree position (BJEU06-1-like: 22 vs. 16). To understand the differences between ORF5 and whole-genome variations, we analyzed amino acid and glycosylation sites of the GP5 protein encoded by ORF5. The results indicated that mutations had occurred at amino acid sites within the antigenic epitopes and functional domains of GP5. Additionally, the prediction of potential N-glycosylation sites identified five locations in GP5: positions 35, 37, 38, 46, and 53. Alterations at these sites could facilitate immune evasion. Our analysis of the ORF5 gene suggests that PRRSV-1 research should not focus solely on ORF5 but rather must consider whole-genome variation, as this may provide insights for vaccine development. In summary, whole-genome studies of PRRSV-1 demonstrated that major recombinant subgroups and genetic evolution align with the current prevalence of BJEU06-1-like strains in China. Analysis of GP5 encoded by ORF5 confirmed the presence of differences between whole-genome and ORF5 data, exhibiting minor discrepancies in both the phylogenetic trees and the level of genetic diversity. Thus, instead of focusing solely on specific regions, whole-genome studies are needed to effectively track variation in PRRSV. This study fills a knowledge gap in our understanding of the prevalence and genetic variation of PRRSV-1 in China, providing crucial insights for developing PRRS control strategies and offering theoretical support for vaccine development.

## Introduction

Porcine reproductive and respiratory syndrome (PRRS), caused by the PRRS virus (PRRSV), is a natural pathogen affecting domestic pigs and wild boar. First identified in the United States in 1987, the virus has persisted as a major threat to the global swine industry for over 3 decades (1). Characteristic clinical manifestations include severe reproductive disorders in pregnant sows (e.g., fetal mummification, stillbirth, and abortion) and respiratory symptoms in piglets, with rapid transmission having been documented in nearly all pig-producing countries (2). PRRSV is an enveloped, single-stranded positive-sense RNA virus featuring a ~ 15 kb genome and spherical virions (3). Its genomic architecture includes a 5′-untranslated region (UTR), a 3′-terminal poly (A) tail, and at least 11 open reading frames (ORFs)– ORF1a and ORF1b encode 16 non-structural proteins (Nsps) spanning ~80% of the genome, while ORF2–ORF7 encode eight structural proteins (4). ORF5 serves as the primary target for molecular surveillance and phylogenetic analysis (5, 6), with its encoded GP5 protein (a major immunogenic structural protein) representing the focal point for vaccine development (7–10). Phylogenetic analysis based on ORF5 gene sequences and global PRRSV classification systems assigns PRRSV-1 into three or four subtypes [subtype 1 (global), subtype 1 (Russian), subtype 2, and subtype 3] (11, 12), while PRRSV-2 comprises 11 lineages (L1–L11) (13). Despite sharing only ~60% nucleotide sequence homology, PRRSV-1 and PRRSV-2 exhibit nearly identical pathogenic mechanisms and transmission patterns (14, 15). PRRSV’s high rates of mutation and recombination are the primary obstacles to disease control, creating an urgent global public health challenge that demands effective prevention strategies. In China, PRRSV-2 predominates through widespread circulation of lineages L8 (HP-PRRSV), L5 (VR-2332), L3 (QYYZ), and L1 (NADC30-like, NADC34-like) (16–21). Concurrently, PRRSV-1 has persisted for decades, with contemporary Chinese isolates clustering within Subtype 1 and forming four principal subgroups: Amervac-like, BJEU06-1-like, HKEU16-like, and NMEU09-like (22, 23). The Chinese strains have historically been understudied due to their low pathogenicity and low detection rates. Recent evidence indicates divergence in virulence among the Chinese PRRSV-1 strains (24). For example, the domestically isolated ZD-1 strain induces fever, pulmonary lesions, and mortality in piglets, suggesting a shift in virulence toward low-to-moderately virulent phenotypes (25). Notably, PRRSV-1 detection has surged, with genetically diverse strains now reported across more than 23 provinces. The southward expansion of BJEU06-1-like strains from northern regions is an emerging concern (26). Persistent PRRSV-1 detection, coupled with high mutation rates and genetic diversity, poses a substantial latent threat to China’s swine industry (19). Critical research gaps persist regarding the pathogenesis and immune mechanisms, as well as vaccine development, resulting in inadequate control strategies and surveillance systems. Consequently, commercial PRRSV-1 vaccines remain rare. China prohibits modified live vaccines (MLVs) due to recombination risks: MLV strains may recombine with endemic field variants (e.g., BJEU06-1-like strains), potentially generating novel variants with enhanced virulence or transmissibility (11, 27). Given these findings, there is an urgent need for enhanced surveillance of the understudied PRRSV-1 strains. A comprehensive analysis of Chinese PRRSV-1 whole-genome sequences, including genetic divergence, diversity patterns, recombination events, and GP5 protein characteristics (amino acid substitutions and N-glycosylation sites), will enhance epidemic monitoring and provide foundational insights for PRRS control strategies.

## Materials and methods

### Genome sequence retrieval and processing

All available Chinese PRRSV-1 complete genomes (*n* = 46; Supplementary Table S2) were retrieved from GenBank (Release 265). The sequences were aligned with MAFFT along with two reference strains (PRRSV-1: M96262.2; PRRSV-2: AY150564.1; Supplementary Table S2), followed by manual trimming of the terminal non-coding regions (28).

### Genotyping and genetic distance analysis

The trimmed sequences were used to construct phylogenetic trees in MEGA7.0.26 using the Kimura two-parameter model and the neighbor-joining method, with 1,000 bootstrap replicates. Genotypic classification was determined based on topological clustering. Pairwise nucleotide p-distances were calculated genome-wide using the same K2P model (29, 30).

### Detection of recombination

Putative recombination events were identified using RDP4, with eight detection algorithms (3seq, BootScan, Chimera, GENECONV, LARD, MaxChi, RDP, and SiScan). Events supported by three or more methods were considered validated. Recombination breakpoints within the ORF1–ORF7 regions were confirmed via Simplot 3.5.1 (reference strain: M96262.2; Supplementary Figure S2). Recombination frequency statistics were computed in Excel 365 (31).

### GP5 protein characterization

ORF5-encoded GP5 protein sequences were analyzed for the following. Sequence homology and variation: alignment via MegAlign (DNASTAR) with the identification of substitutions/deletions (32). N-glycosylation sites: prediction using NetNGlyc 1.0 (threshold: > 0.5) at the Asn-X-Ser/Thr motifs (33).

## Results

### Phylogenetic analysis of whole genomes of Chinese PRRSV-1

**Figure 1:**
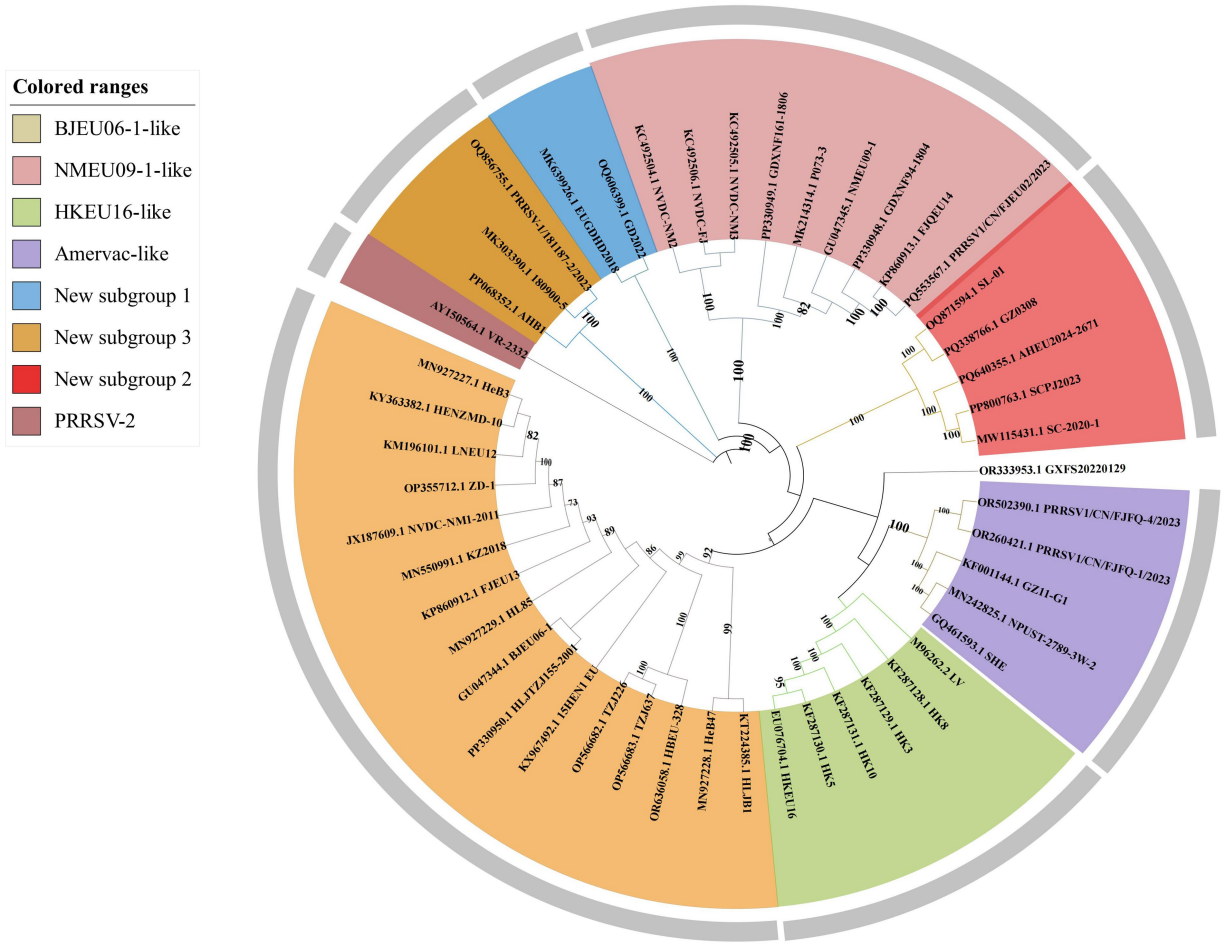
Phylogenetic tree of the complete genome of PRRSV-1 in China.

A phylogenetic analysis of 46 complete genomes of Chinese PRRSV-1 and representative strains (PRRSV-1: LV; PRRSV-2: VR-2332) demonstrated that all Chinese isolates belonged to subtype 1, forming four subgroups: BJEU06-1-like, Amervac-like, HKEU16-like, and NMEU09-1-like (Figure 1). Among these, BJEU06-1-like was the predominant subgroup (16 strains in the whole-genome vs. 22 in the ORF5 analysis). Additionally, we identified several small novel subgroups designated as new subgroups 1, 2, and 3, while also detecting a new strain, GD2022, within these subgroups.

**Figure 2:**
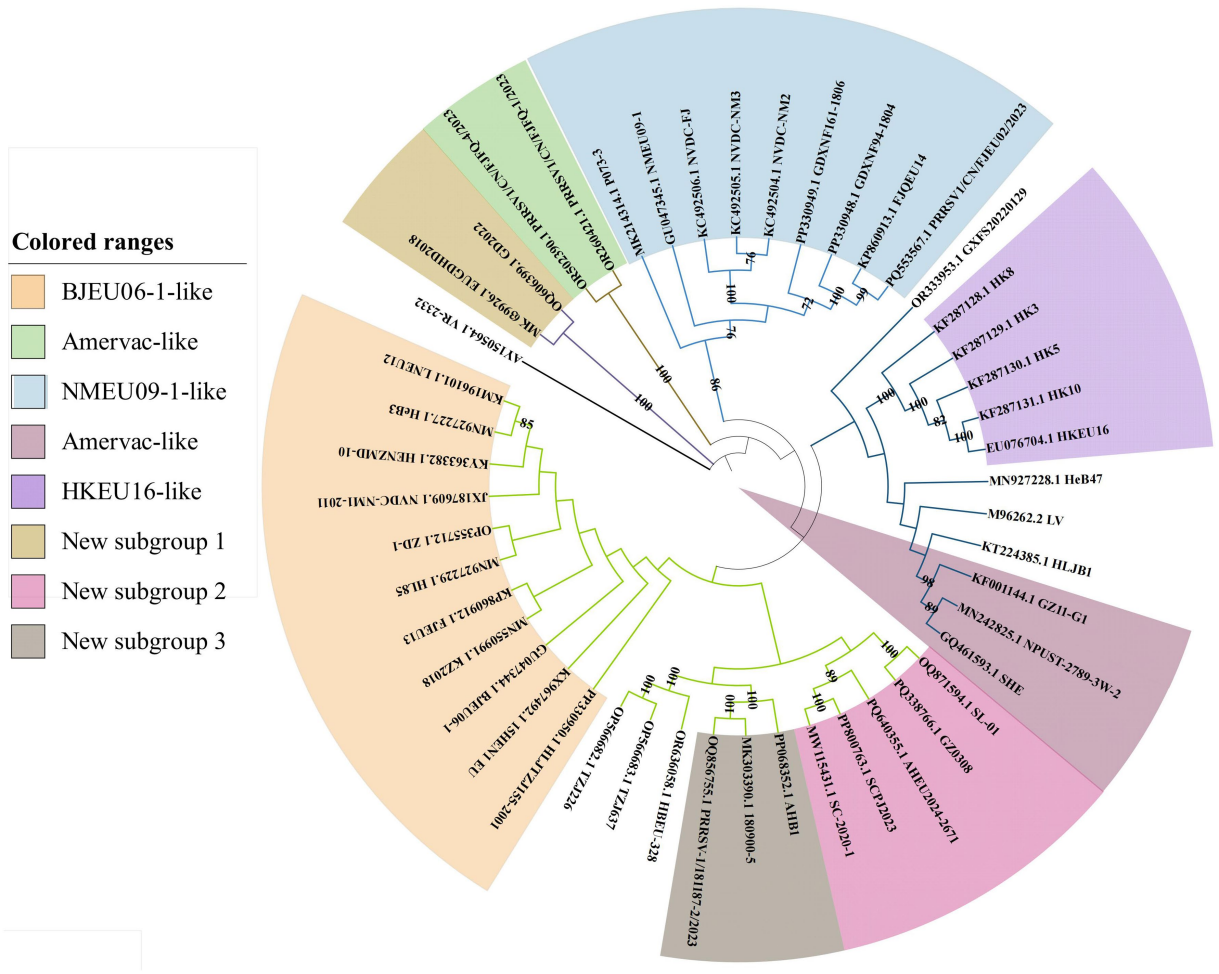
Phylogenetic tree of ORF5 Chinese PRRSV-1.

There were several notable differences between the whole-genome and ORF5 phylogenies (Figure 2). Vaccine strains (SHE, NPUST-2789-3 W-2) clustered within Amervac-like in the whole-genome trees but grouped with field strains (HeB47, LV, HLJB1, and GZ11-G1) in the ORF5-based analysis. New subgroups 2 and 3 formed separate branches in the whole-genome trees, but clustered within BJEU06-1-like in the ORF5 phylogenies. Strain GXFS20220129 comprised an independent branch in both analyses. These differences indicated that whole-genome analysis provides a more detailed characterization of the variation in PRRSV-1. The continuous emergence of new strains and variants necessitates more intensive research on PRRSV-1 in China, and whole-genome analysis would better capture the epidemiological trends of the virus, thereby improving the control of PRRS.

### Genetic diversity of Chinese PRRSV-1 whole genomes

The observed variation in whole genomes of PRRSV-1 reflects inter-subgroup divergence. We calculated nucleotide genetic distances for the complete genome sequences to understand evolutionary relationships among the PRRSV-1 subgroups. Intra-subgroup genetic distances were lowest in new subgroup 1 (0.005), while BJEU06-1-like (0.118), NMEU09-1-like (0.127), and new subgroup 2 (0.139) exhibited greater distances than the other subgroups, consistent with their status as predominant epidemic subgroups (Table 1). Inter-subgroup genetic distances showed greater divergence between BJEU06-1-like and NMEU09-1-like (0.189), new subgroups 1 (0.190), 2 (0.191), and 3 (0.207). BJEU06-1-like had the least distance from the prototype strain LV (0.110). Strain GXFS20220129 was more distant than NMEU09-1-like (0.193), new subgroup 1 (0.191), new subgroup 3 (0.205), and BJEU06-1-like (0.164). New subgroup 3 exhibited significant divergence from all other subgroups (0.173 ~ 0.220). Greater genetic distances are correlated with increased genetic diversity. Considerable genetic variation exists among Chinese PRRSV-1 subgroups, particularly in phylogenetically distinct lineages GXFS20220129 and new subgroup 3, both of which exhibit diversity across subgroups. This variability complicates the control of PRRSV-1 in China and requires attention (Table 2).

**Table 1:** Genetic distance of nucleotides within subgroups.

Genotype	Subgroup	
Type 1	BJEU06-1-like	0.118
	Amervac-like	0.076
	HKEU16-like	0,051
	NMEU09-1-like	0.127
	New subgroup 1	0.005
	New subgroup 2	0.139
	New subgroup 3	0.023

**Table 2:** Genetic distances of nucleotides between subgroups.

Subgroup	BJEU06-1-like	Amervac-like	HKEU16-like	NMEU09-1-like	New subgroup 1	New subgroup 2	New subgroup 3	M96262.2
Amervac-like	0.153							
HKEU16-like	0.156	0.139						
NMEU09-1-like	0.189	0.171	0.180					
New subgroup 1	0.190	0.168	0.181	0.205				
New subgroup 2	0.191	0.178	0.176	0.207	0.209			
New subgroup 3	0.207	0.196	0.194	0.218	0.220	0.196		
M96262.2	0.110	0.089	0.092	0.151	0.145	0.089	0.173	
OR333953.1	0.164	0.152	0.155	0.193	0.191	0.152	0.205	0.130

### Recombination analysis of Chinese PRRSV-1 whole genomes

**Figure 3:**
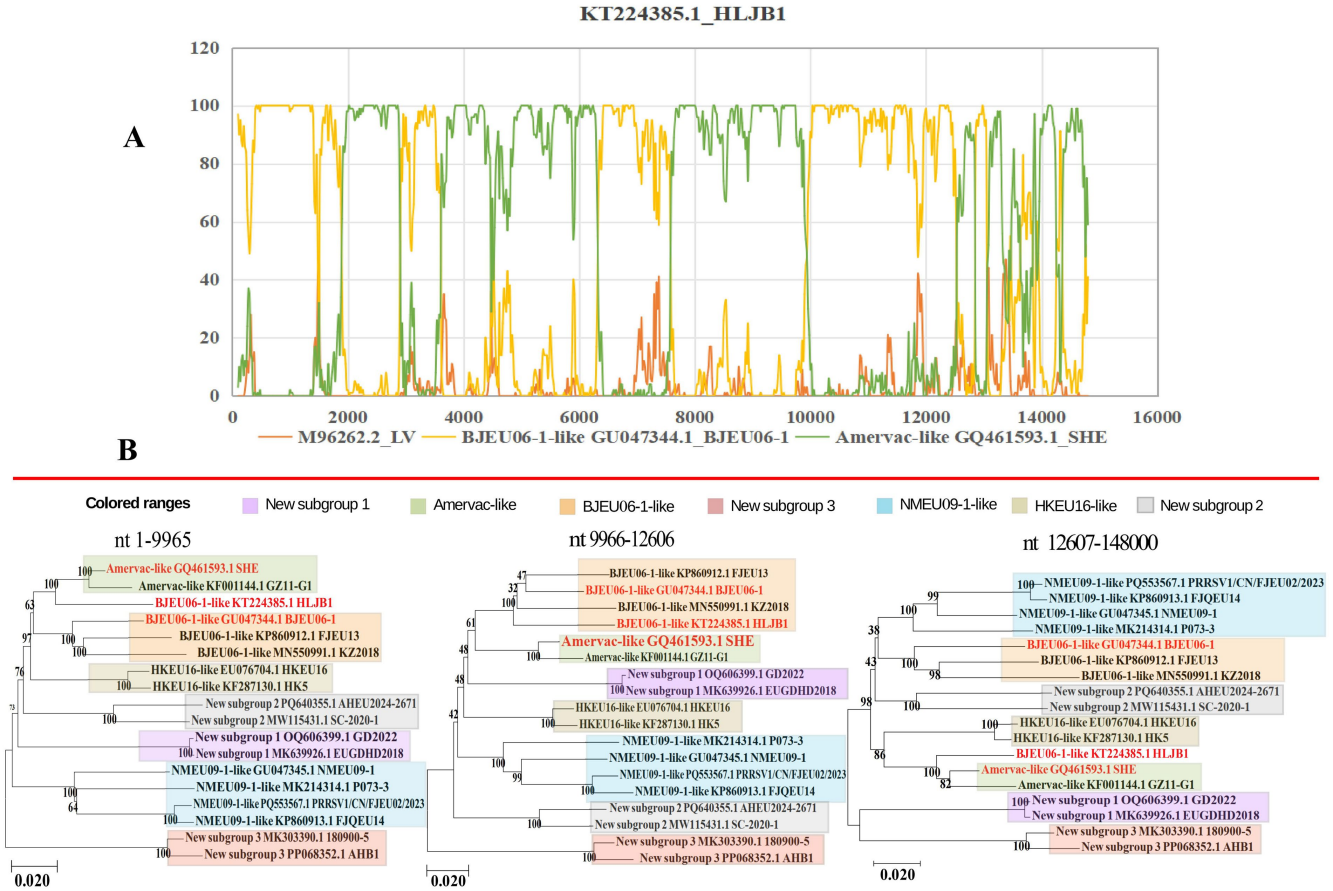
Reorganization analysis results of HLJB1 strain.

Recombination is recognized as a key driving force of high mutation rates and genetic diversity in PRRSV genomes (11, 34–36). We analyzed the recombination events in PRRSV-1 whole genomes, identifying 32 recombination events, of which 9 intra-subgroup events (mainly BJEU06-1-like) and 23 inter-subgroup events (primarily BJEU06-1-like + Amervac-like, *n* = 7). A recombination analysis of strain HLJB1 using Simplot 3.5.1 and RDP4 confirmed a recombination event with breakpoints at nucleotides 9,966–12,606 nt (Figure 3A). Subsequent segmented phylogenetic analysis of recombinant regions in this strain (HLJB1) identified both major and minor parental lineages belonging to PRRSV-1 (Figure 3B). In addition, inter-subgroup recombination occurred between the vaccine and field strains. For the vaccine strain PRRSV1-CN-FJFQ-1-2023 (Amervac-like), the major parental strain was GZ11-G1 (Amervac-like), and the minor parental strain was NVDC-FJ (NMEU09-1-like). A recombination hotspot analysis showed frequent breakpoints in the ORF1a and ORF1b genes (Supplementary Figure S2).

Novel subgroups exhibited inter-subgroup recombination: strain MK303390.1 (new subgroup 3) had PRRSV1-CN-FJEU02-2023 (NMEU09-1-like) as the major parent and LV as the minor parent. Recombination occurred between two of the new subgroups: strain SCPJ2023 (new subgroup 2) had EUGDHD2018 (new subgroup 1) as the major parent and SL-01 (new subgroup 2) as the minor parent. These findings indicate that recombination has contributed to the genetic variation and phylogenetic differences among subgroups, thereby explaining, to some extent, the recent spread of PRRSV-1 in China. We observed a shift from simple intra-subgroup recombination to complex inter-subgroup recombination in Chinese PRRSV-1, a factor that complicates the potential control of the disease (Supplementary Table S1).

### ORF5 nucleotide and amino acid homology analysis

ORF5 is routinely used for molecular epidemiological surveillance and vaccine development. The genetic diversity analysis of ORF5 has provided insights into the evolutionary trends of PRRSV-1 in China. We therefore examined the nucleotide and amino acid homology of ORF5 genes from Chinese PRRSV-1 whole genomes. The results showed that the nucleotide homology of PRRSV-1 ORF5 genes ranged from 77.7 to 100%, with amino acid homology ranging from 77.2 to 100%. Within HKEU16-like strains, the level of nucleotide homology (92.4–99.7%) indicated higher conservation and lower genetic variation compared to other subgroups. Chinese PRRSV-1 sequences shared 81.8–94.6% homology with European reference strain M96262.2 (LV), suggesting limited genetic diversity between regional and European strains. Notably, intra-subgroup homology varied substantially, with BJEU06-1-like, NMEU09-1-like, and new subgroup 2 exhibiting higher variability. The amino acid homology was elevated in new subgroup 1 (98.5%), new subgroup 3 (92.6–98%), and HKEU16-like (92.1–99.5%), patterns consistent with the nucleotide results. Inter-subgroup comparisons revealed greater genetic divergence, with nucleotide and amino acid homology in the ranges 77.7–100% and 77.2–100%, respectively (Table 3).

**Table 3:** ORF5 nucleotide and amino acid homology analysis.

Subgroup	Nucleotide ORF5	Amino acid ORF5
New subgroup 1	99.2	98.5
New subgroup 2	81.7–99.8	81.2–99.5
New subgroup 3	94.6–99.2	92.6–98
Amervac-like	88.4–96.7	82.7–97.5
HKEU16-like	92.4–99.7	92.1–99.5
NMEU09-1-like	85.1–100	85.6–100
BJEU06-1-like	83.3–97	81.7–99.5
M96262.2	81.8–94.6	81.7–93.6
OR333953.1	80.2–89.9	81.2–88.6
PRRSV-1	77.7–100	77.2–100

### GP5 protein amino acid substitution analysis

**Figure 4:**
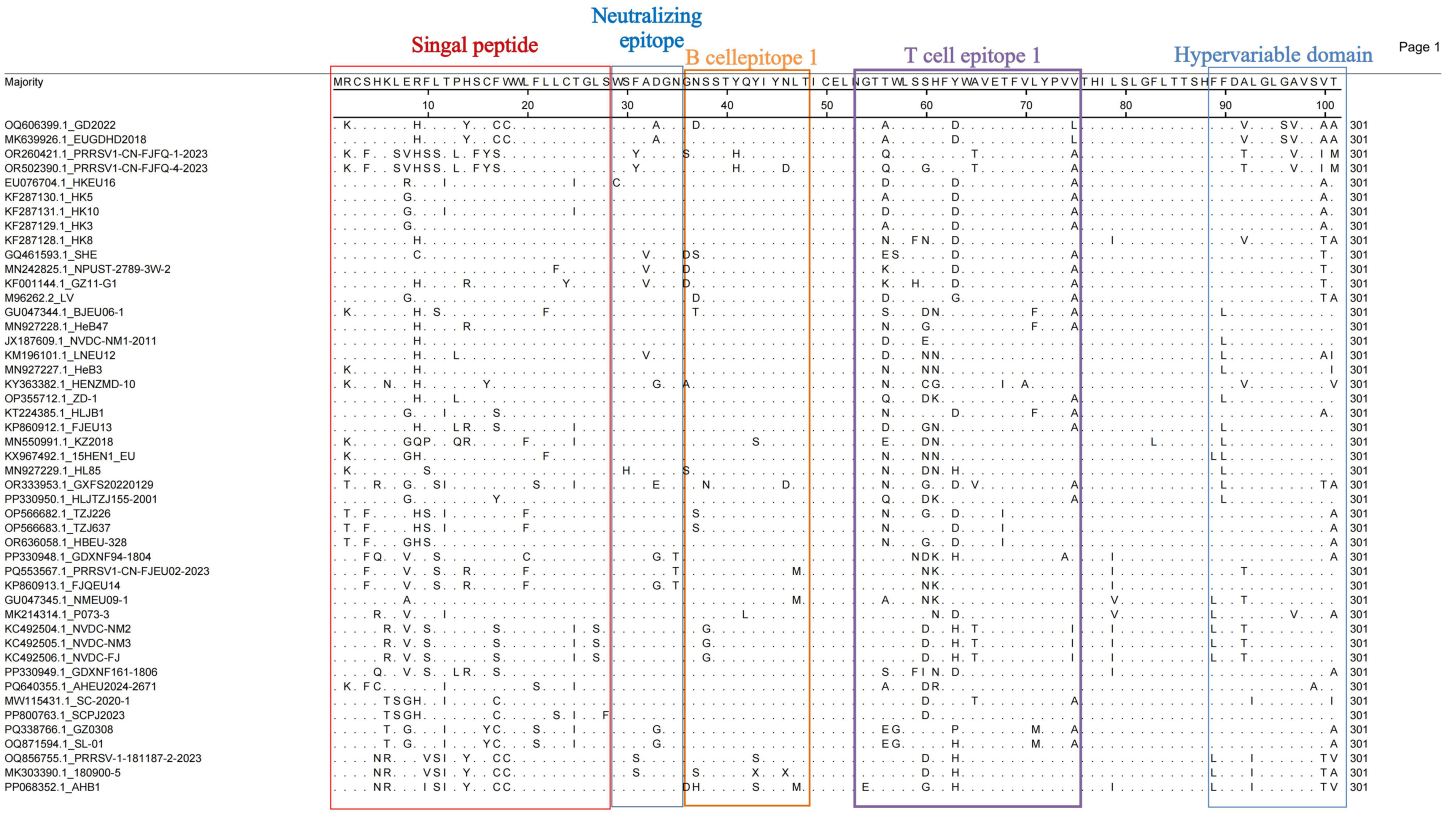
Amino acid site analysis of Chinese PRRSV-1 GP5 protein (Amino acids 1 to 101).

**Figure 5:**
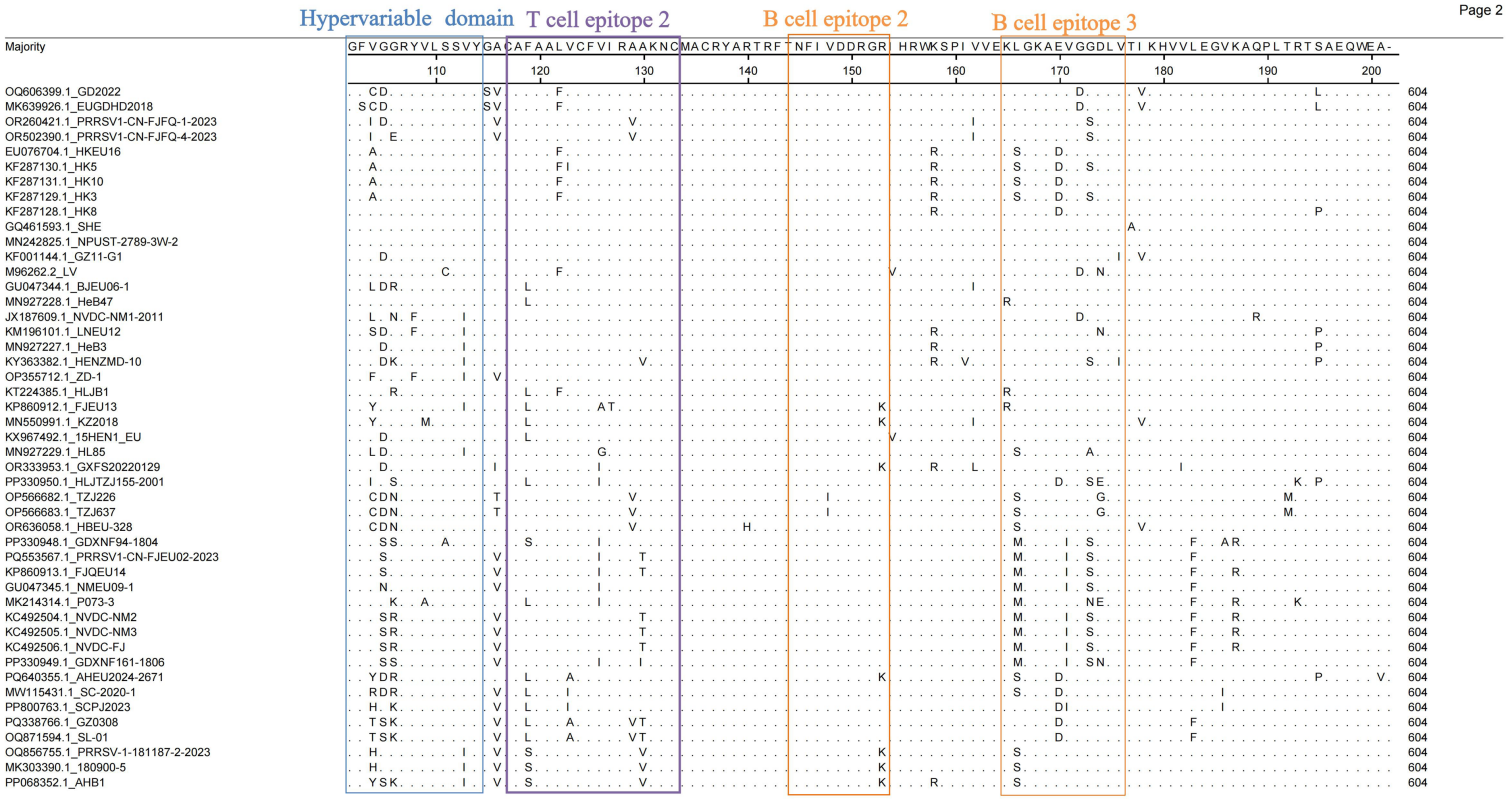
Amino acid site analysis of Chinese PRRSV-1 GP5 protein (Amino acids 102 to 201).

Given the high variability of the ORF5-encoded GP5 protein, analyzing GP5 genetic variation has become essential for developing novel PRRSV vaccines and controlling viral transmission. We analyzed the amino acid substitution sites in GP5 from 47 PRRSV-1 ORF5 genes using MegAlign (DNASTAR) to characterize the GP5 composition for improved control of PRRSV-1. The GP5 protein comprises 201 amino acids encoded by 603 nucleotides. Structural prediction revealed a signal peptide, a hypervariable region, and multiple B-cell and T-cell epitopes. In the signal peptide region, position 9 Arg (R) was mutated to His (H) in most strains (Figure 4). Cys^24^ (C24) and ^29^WSFADGN^35^ regions were correlated with neutralizing antibody epitopes, where C24 was highly conserved except for a mutation in GZ11-G1 (Figure 5). Neutralizing epitopes play critical roles in anti-PRRSV responses; we observed substitutions at the ^29^W-^35^N epitopes, including Trp^29^ → Cys (C) in HKEU16. At B-cell epitope position 37 Asn (N), the GD2022 and LV strains exhibited Asn^37^ → Asp (D) mutations, BJEU06-1 had Asn^37^ → Thr (T), and SHE/TZJ226/TZJ637/180900–5 exhibited Asn^37^ → Ser (S). In addition, mutations of Val^32^ (V) occurred in the Amervac-like strains (SHE, GZ11-G1, NPUST-2789-3 W-2) and the BJEU06-1-like strain LNEU12 (Table 3).

### Analysis of potential N-glycosylation sites in the GP5 protein

As a major structural protein inducing protective antibodies, alterations in the glycosylation sites of the PRRSV-1 GP5 protein will impact its immunological efficacy. We thus investigated potential glycosylation sites for the protein. The analysis revealed five potential N-glycosylation sites at amino acid positions 35, 37, 38, 46, and 53 (Table 4). Consistent with the patterns of amino acid variation, mutations occurred at position 37 N in strains GD2022, LV, SHE, BJEU06-1, TZJ226, TZJ637, 180,900–5, and AHB1. At position 35 N—an antigenic epitope site—amino acid additions were observed in SHE, BJEU06-1, TZJ226, TZJ637, and 180,900–5. Concurrently, position 46 N exhibited deletions in GXFS20220129, PRRSV1-CN-FJFQ-4-2023, and 180,900–5 (Table 4).

**Table 4:** Analysis of potential N-glycosylation sites in the GP5 protein.

Strains	Potential N-glycosylation sites				
	35	37	38	46	53
GD2022				NLT	NGT
EUGDHD2018		NSS		NLT	NGT
PRRSV1-CN-FJFQ-1-2023		NSS		NLT	NGT
PRRSV1-CN-FJFQ-4-2023		NSS			NGT
HKEU16		NSS		NLT	NGT
HK5		NSS		NLT	NGT
HK10		NSS		NLT	NGT
HK3		NSS		NLT	NGT
HK8		NSS		NLT	NGT
SHE	NDS			NLT	NGT
NPUST-2789-3 W-2		NSS		NLT	NGT
GZ11-G1		NSS		NLT	NGT
LV				NLT	NGT
BJEU06-1	NGT			NLT	NGT
HeB47		NSS		NLT	NGT
NVDC-NM1-2011		NSS		NLT	NGT
LNEU12		NSS		NLT	NGT
HeB3		NSS		NLT	NGT
HENZMD-10		NSS		NLT	NGT
ZD-1		NSS		NLT	NGT
HLJB1		NSS		NLT	NGT
FJEU13		NSS		NLT	NGT
KZ2018		NSS		NLT	NGT
15HEN1_EU		NSS		NLT	NGT
HL85		NSS		NLT	NGT
GXFS20220129		NNS	NST		NGT
HLJTZJ155-2001		NSS		NLT	NGT
TZJ226	NGS			NLT	NGT
TZJ637	NGS			NLT	NGT
HBEU-328		NSS		NLT	NGT
GDXNF94-1804		NSS		NLT	
PRRSV1-CN-FJEU02-2023		NSS		NMT	NGT
FJQEU14		NSS		NLT	
NMEU09-1		NSS		NMT	NGT
P073-3		NSS		NLT	NGT
NVDC-NM2		NGS		NLT	NGT
NVDC-NM3		NGS		NLT	NGT
NVDC-FJ		NGS		NLT	NGT
GDXNF161-1806		NSS		NLT	NGT
AHEU2024-2671		NSS		NLT	NGT
SC-2020-1		NSS		NLT	
SCPJ2023		NSS		NLT	
GZ0308		NSS		NLT	NGT
SL-01		NSS		NLT	NGT
PRRSV-1-181187-2-2023		NSS		NLT	NGT
180,900–5	NGS				NGT
AHB1				NMT	

## Discussion

The increasing prevalence of PRRSV, driven by its characteristically high mutation and recombination rates (37, 38), coincides with the rise in PRRSV-1 detection across China. Concurrently, effective prevention and control strategies remain critically underdeveloped. The investigation of epidemiological trends and geographic distribution of PRRSV-1 in China provides essential data to inform the development of vaccines and efforts to contain the epidemic. Genetic differentiation reflects inter-population genetic variation and the dynamics of disease prevalence. Our genetic differentiation analysis of 46 Chinese PRRSV-1 whole-genome strains, together with the reference strain VR-2332 and thorough phylogenetic analysis, revealed that contemporary Chinese PRRSV-1 primarily clustered into four subgroups: BJEU06-1-like, Amervac-like, HKEU16-like, and NMEU09-1-like (23). Among these, BJEU06-1-like represented the dominant subgroup, followed by NMEU09-1-like. The identification of novel branches and emerging strains (e.g., GD2022) (11, 39) indicates increasing complexity in PRRSV-1 epidemiology within China, with substantial genetic variation necessitating heightened vigilance. Since the ORF5 gene serves as the primary diagnostic target for PRRSV detection, we constructed a phylogenetic tree using ORF5 sequences derived from whole genomes. Notably, discrepancies emerged between the ORF5-based and whole-genome phylogenies: strains classified as Amervac-like (SHE, NPUST-2789-3 W-2, GZ11-G1) in the whole-genome analysis clustered with LV and BJEU06-1-like strains (HeB47, HLJB1) in the ORF5 tree. Previous studies have documented recombination between HeB47/HLJB1 and Amervac-like strains (23, 40). We attribute this phylogenetic convergence to high ORF5 sequence homology and inter-strain recombination events. The new subgroups 2 and 3 clustered within BJEU06-1-like in the ORF5 tree, whereas new subgroup 1 formed a distinct branch in both phylogenies, suggesting potential inter-subgroup recombination. The continuous emergence of novel branches and strains demonstrates the persistent evolution of the virus in China, posing a significant risk of viral enhancement that demands increased surveillance. Genetic diversity reflects viral divergence and the potential for mutation. Analysis of whole-genome genetic diversity revealed elevated diversity within the prevalent subgroups BJEU06-1-like, NMEU09-1-like, and Amervac-like, which may explain their epidemiological success. Novel branches also exhibited high diversity, indicating potential for future dominance and warranting close monitoring. Motivated by the observed phylogenetic discrepancies, we conducted nucleotide and amino acid homology analyses of the ORF5 gene. The results confirmed high intra-subgroup diversity within BJEU06-1-like, NMEU09-1-like, and new subgroup 3, consistent with the whole-genome findings and indicative of heightened mutability. The intricate relationship between genetic diversity and variation warrants increased attention. While the ORF5 phylogeny partially diverged from the whole-genome analysis, homology assessment alone cannot establish causality. Frequent recombination of PRRSV-1 drives continuous variation among strains and significantly facilitates the emergence of novel strains. The recombination analysis of 46 whole-genome sequences and reference strain LV identified 32 recombination events, with inter-subgroup recombination exceeding intra-subgroup events. This indicates an epidemiological shift from single-subgroup dominance to co-circulation among multiple subgroups in China. BJEU06-1-like (12 events) and NMEU09-1-like (seven events) were the predominant recombinant subgroups, aligning with their status as major epidemic strains. Recombination is thus a key mechanism driving their dominance, high level of diversity, and adaptability. Beyond confirming the documented recombination between vaccine strains (e.g., Amervac-like) and field strains such as HLJB1/HeB47 (23, 40), we detected additional vaccine-field recombination events. Notably, vaccine strain Amervac-like_GQ461593.1_SHE exhibited frequent recombination with field strains—a finding with potential implications for vaccine design that warrants further vigilance. Frequent recombination between BJEU06-1-like and Amervac-like strains necessitates caution to prevent the emergence of epidemic strains and co-infections involving vaccine and field viruses. Strains showing phylogenetic discordance (Amervac-like: SHE, NPUST-2789-3 W-2, GZ11-G1; BJEU06-1-like: HeB47, HLJB1) demonstrated mutual recombination, which explains their ORF5 clustering. The novel branch (11), arising exclusively from inter-subgroup recombination, exhibits high variability and potential epidemic significance, thereby demanding prioritized surveillance. Collectively, these findings reveal increasingly complex transmission dynamics and growing challenges to controlling PRRSV-1 in China. There was partial discordance between whole-genome and ORF5 phylogenies; GD2022 formed a distinct branch in both trees without evidence of recombination, suggesting an emerging lineage (39). The disparity in detected recombination events (32 whole-genome vs. one ORF5) highlights the inadequacy of ORF5 as a full-genome surrogate. Nevertheless, ORF5 remains crucial for surveillance due to its key roles in neutralizing antibody responses, receptor binding, immune evasion (41), and as a primary subunit vaccine target. Substitutions, deletions, and insertions drive PRRSV evolution (42), with variation in the GP5 protein concentrated in the signal peptide as well as neutralizing and non-neutralizing epitope regions (43). Further GP5 amino acid analysis revealed that GD2022’s B-cell epitope mutation (33A) significantly reduces neutralizing antibody titers against vaccine strains and compromises cross-protection (39). Position C24 is highly conserved in PRRSV-1 genomes, with mutation observed only in GZ11-G1 (44), demonstrating intra-subgroup amino acid heterogeneity and highlighting viral complexity. Detailed characterization of the GP5 amino acid distribution is vital for vaccine design (45) and understanding the patterns of viral pathogenicity and transmission (46). Predicting N-glycosylation sites offers insights into the evolution and regulation of PRRSV-1, as glycosylation modulates immune recognition (47). The amino acid acquisitions at position 35 N in strains SHE, BJEU06-1, TZJ226, TZJ637, and 180,900–5 may have contributed to the prevalence of BJEU06-1-like/NMEU09-1-like. Interestingly, GXFS20220129 possesses a unique acquisition at 38 N, potentially altering the virulence of the strain, explaining its phylogenetic divergence, and signaling an emerging risk that demands specific attention. In summary, these findings have advanced our understanding of the prevalence, genetic diversity, recombination patterns, and divergent amino acid sites of PRRSV-1 in China. Future research must prioritize whole-genome sequencing over exclusive reliance on ORF5, as single-gene analysis cannot fully capture epidemiological complexity. This study provides critical insights for developing effective PRRS prevention and control strategies to mitigate viral transmission.

## Data Availability

The datasets presented in this study can be found in online repositories. The names of the repository/repositories and accession number(s) can be found in the article/Supplementary material.
