# Supplementary material for: Genetic evolution, epidemic trends, and recombination dynamics of PRRSV-1 in China
Source: Front Vet Sci. 2025 Aug 5;12:1632917. doi: 10.3389/fvets.2025.1632917 (PMC12363365; doi:10.3389/fvets.2025.1632917)
Supplement: Supplementary file 1 [file Data_Sheet_1.docx]

Supplementary Table S1. Recombination Events in the Whole Genome of 47 PRRSV-1 Strains

| recombination sequence | Main parental sequences | Sequence of secondary parents | The breakpoint position/bp | Algorithm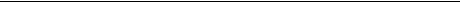  RDP GENECONV Bootscan Maxchi Chimaera SiSscan 3Seq | | | | | | |
| --- | --- | --- | --- | --- | --- | --- | --- | --- | --- | --- |
| BJEU06-1-like JX187609.1_NVDC-NM1-2011 | BJEU06-1-like MN927227.1_HeB3 | BJEU06-1-like GU047344.1_BJEU06-1 | 355~2362 | ﹢ | ﹢ | ﹢ | ﹢ | ﹢ | ﹢ | ﹢ |
| BJEU06-1-like JX187609.1_NVDC-NM1-2011 | BJEU06-1-like MN927227.1_HeB3 | BJEU06-1-like GU047344.1_BJEU06-1 | 6820~8980 | ﹢ | ﹢ | ﹢ | ﹢ | ﹢ | ﹢ | ﹢ |
| NMEU09-1-like KC492504.1_NVDC-NM2 | NMEU09-1-like MK214314.1_P073-3 | BJEU06-1-like GU047344.1_BJEU06-1 | 696~3236 | ﹢ | ﹢ | ﹢ | ﹢ | ﹢ | ﹢ | ﹢ |
| New subgroup 2-OQ871594.1_SL-01 | New subgroup 2-PQ338766.1_GZ0308 | M96262.2_LV | 2514~2666 | ﹢ | ﹢ | ﹢ | ﹢ | ﹢ | ﹢ | - |
| HKEU16-like EU076704.1_HKEU16 | HKEU16-like KF287131.1_HK10 | M96262.2_LV | 8982~9410 | ﹢ | ﹢ | ﹢ | ﹢ | ﹢ | ﹢ | - |
| BJEU06-1-like PP330950.1_HLJTZJ155-2001 | M96262.2_LV | BJEU06-1-like MN927229.1_HL85 | 158~1640 | ﹢ | ﹢ | ﹢ | ﹢ | ﹢ | ﹢ | - |
| HKEU16-like EU076704.1_HKEU16 | HKEU16-like KF287130.1_HK5 | HKEU16-like KF287131.1_HK10 | 12914~1908 | ﹢ | ﹢ | ﹢ | ﹢ | ﹢ | ﹢ | ﹢ |
| BJEU06-1-like KT224385.1_HLJB1 | Amervac-like GQ461593.1_SHE | BJEU06-1-like GU047344.1_BJEU06-1 | 9966~12606 | ﹢ | - | ﹢ | ﹢ | ﹢ | - | - |
| BJEU06-1-like KT224385.1_HLJB1 | Amervac-like GQ461593.1_SHE | BJEU06-1-like KX967492.1_15HEN1_EU | 114~1818 | ﹢ | - | ﹢ | ﹢ | ﹢ | ﹢ | - |
| BJEU06-1-like KT224385.1_HLJB1 | Amervac-like GQ461593. 1_SHE | BJEU06-1-like GU047344.1_BJEU06-1 | 6382~7556 | ﹢ | - | ﹢ | ﹢ | ﹢ | ﹢ | ﹢ |

Continued table

| recombination sequence | Main parental sequences | Sequence of secondary parents | The breakpoint position/bp | Algorithm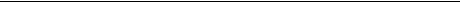  RDP GENECONV Bootscan Maxchi Chimaera SiSscan 3Seq | | | | | | |
| --- | --- | --- | --- | --- | --- | --- | --- | --- | --- | --- |
| BJEU06-1-like GU047344.1_BJEU06-1 | M96262.2_LV | NMEU09-1-like KC492506.1_NVDC-FJ | 12882~13334 | ﹢ | - | ﹢ | - | ﹢ | - | - |
| BJEU06-1-like MN550991.1_KZ2018 | Unknown (BJEU06-1-like KP860912.1_FJEU13) | BJEU06-1-like KY363382.1_HENZMD-10 | 14527~1732 | ﹢ | - | ﹢ | ﹢ | ﹢ | ﹢ | - |
| BJEU06-1-like KT224385.1_HLJB1 | Amervac-like MN242825.1_NPUST-2789-3W-2 | BJEU06-1-like JX187609.1_NVDC-NM1-2011 | 2934~3532 | - | - | - | ﹢ | ﹢ | ﹢ | - |
| HKEU16-like KF287131.1_HK10 | HKEU16-like KF287128.1_HK8 | NMEU09-1-like PQ553567.1_PRRSV1-CN-FJEU02-2023 | 12860~13252 | ﹢ | - | ﹢ | ﹢ | ﹢ | - | - |
| HKEU16-like KF287129.1_HK3 | HKEU16-like KF287128.1_HK8 | NMEU09-1-like PQ553567.1_PRRSV1-CN-FJEU02-2023 | 11611~12260 | ﹢ | - | ﹢ | ﹢ | ﹢ | - | - |
| NMEU09-1-like PQ553567.1_PRRSV1-CN-FJEU02-2023 | NMEU09-1-like KP860913.1_FJQEU14 | NMEU09-1-like GU047345.1_NMEU09-1 | 12390~12484 | ﹢ | + | ﹢ | - | - | - | - |
| BJEU06-1-like MN550991.1_KZ2018 | Unknown (BJEU06-1-like KP860912.1_FJEU13) | BJEU06-1-like KY363382.1_HENZMD-10 | 2334~6281 | ﹢ | - | - | ﹢ | ﹢ | + | + |
| HKEU16-like KF287130.1_HK5 | HKEU16-like KF287131.1_HK10 | HKEU16-like KF287129.1_HK3 | 1333~2688 | ﹢ | - | ﹢ | ﹢ | ﹢ | + | - |
| Amervac-like OR260421.1_PRRSV1-CN-FJFQ-1-2023 | Amervac-like KF001144.1_GZ11-G1 | NMEU09-1-like KC492506.1_NVDC-FJ | 12044~12697 | ﹢ | - | ﹢ | ﹢ | ﹢ | - | - |
| BJEU06-1-like MN927228.1_HeB47 | Unknown (M96262.2_LV) | Amervac-like MN242825.1_NPUST-2789-3W-2 | 12568~14886 | - | - | - | ﹢ | ﹢ | + | - |

Continued table

| recombination sequence | Main parental sequences | Sequence of secondary parents | The breakpoint position/bp | Algorithm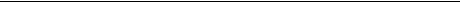  RDP GENECONV Bootscan Maxchi Chimaera SiSscan 3Seq | | | | | | |
| --- | --- | --- | --- | --- | --- | --- | --- | --- | --- | --- |
| BJEU06-1-like MN550991.1_KZ2018 | Unknown (BJEU06-1-like GU047344.1_BJEU06-1) | BJEU06-1-like KY363382.1_HENZMD-10 | 9926~10908 | ﹢ | - | ﹢ | ﹢ | ﹢ | + | - |
| BJEU06-1-like PP330950.1_HLJTZJ155-2001 | BJEU06-1-like MN927227.1_HeB3 | NMEU09-1-like PP330948.1_GDXNF94-1804 | 11475~11794 | ﹢ | - | ﹢ | ﹢ | ﹢ | - | - |
| M96262.2_LV | NMEU09-1-like GU047345.1_NMEU09-1 | New subgroup 3-OQ856755.1_PRRSV-1-181187-2-2023 | 5021~6468 | ﹢ | - | ﹢ | ﹢ | ﹢ | + | - |
| BJEU06-1-like MN927228.1_HeB47 | BJEU06-1-like GU047344.1_BJEU06-1 | Amervac-like GQ461593.1_SHE | 7538~9973 | - | - | - | ﹢ | ﹢ | + | - |
| New subgroup 3-MK303390.1_180900-5 | NMEU09-1-like PQ553567.1_PRRSV1-CN-FJEU02-2023 | M96262.2_LV | 3648~4880 | ﹢ | - | ﹢ | ﹢ | - | + | - |
| M96262.2_LV | BJEU06-1-like OR636058.1_HBEU-328 | Unknown (BJEU06-1-like GU047344.1_BJEU06-1) | 614~965 | ﹢ | - | ﹢ | - | - | + | - |
| New subgroup 2-PP800763.1_SCPJ2023 | New subgroup 1-MK639926.1_EUGDHD2018 | New subgroup 2-OQ871594.1_SL-01 | 5552~6114 | ﹢ | - | - | ﹢ | ﹢ | + | - |
| BJEU06-1-like KM196101.1_LNEU12 | BJEU06-1-like KP860912.1_FJEU13 | HKEU16-like KF287128.1_HK8 | 7120~7308 | ﹢ | - | ﹢ | - | - | + | - |
| HKEU16-like EU076704.1_HKEU16 | HKEU16-like KF287130.1_HK5 | HKEU16-like KF287131.1_HK10 | 9716~10338 | ﹢ | + | ﹢ | + | + | + | - |

Continued table

| recombination sequence | Main parental sequences | Sequence of secondary parents | The breakpoint position/bp | Algorithm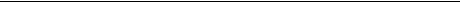  RDP GENECONV Bootscan Maxchi Chimaera SiSscan 3Seq | | | | | | |
| --- | --- | --- | --- | --- | --- | --- | --- | --- | --- | --- |
| Amervac-likeGQ461593.1_SHE | Unknown (BJEU06-1-like JX187609.1_NVDC-NM1-2011) | BJEU06-1-like MN927228.1_HeB47 | 1788~ 2362 | ﹢ | - | - | + | + | + | - |
| BJEU06-1-like MN927228.1_HeB47 | BJEU06-1-like GU047344.1_BJEU06-1 | Amervac-like GQ461593.1_SHE | 3551~4915 | ﹢ | - | ﹢ | + | + | + | - |
| HKEU16-like KF287131.1_HK10 | HKEU16-like EU076704.1_HKEU16 | BJEU06-1-like MN927228.1_HeB47 | 6252~ 6432 | ﹢ | + | - | + | - | - | - |

Note: + indicates that recombination sites were detected; - indicates that no recombination sites were detected

**A**
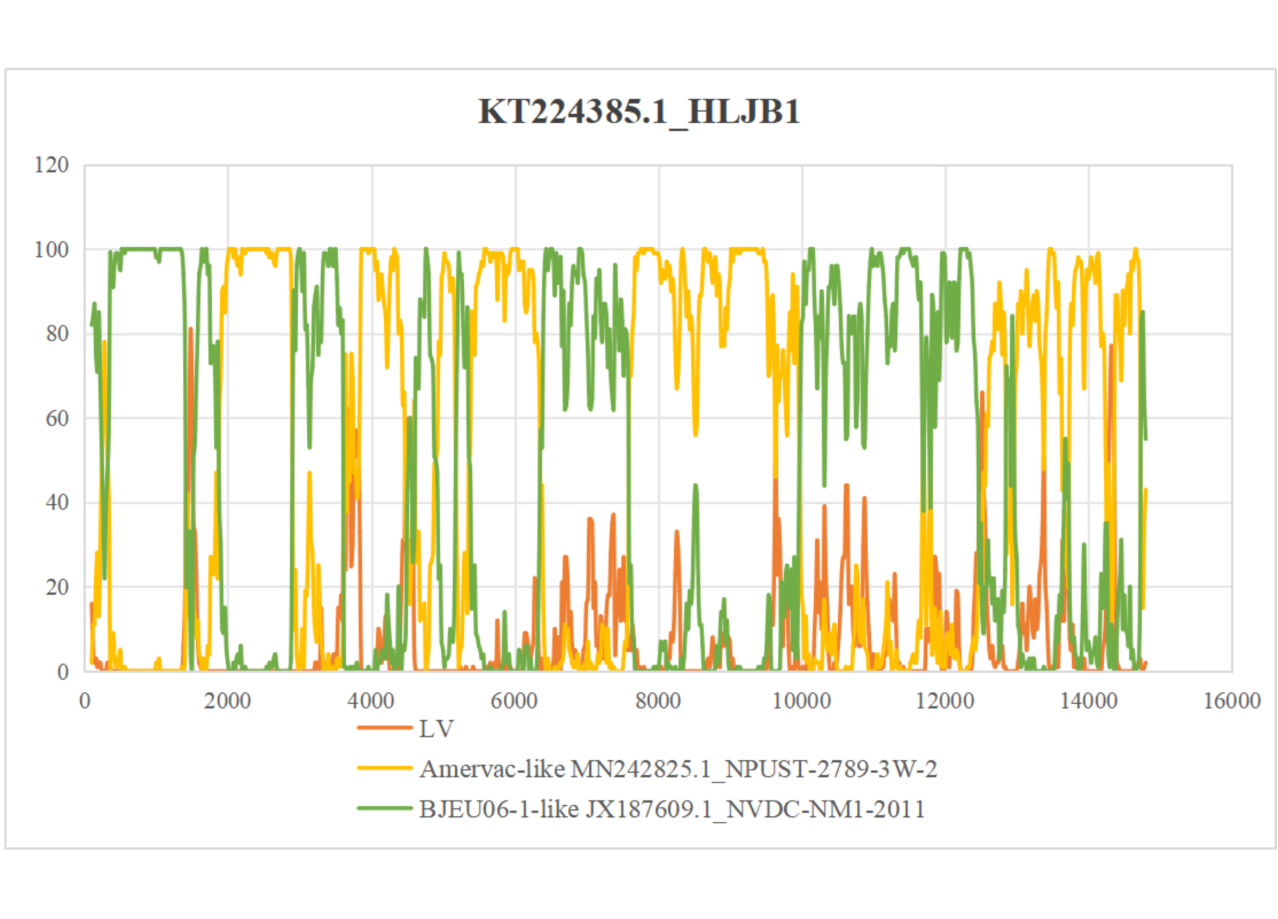


**B**


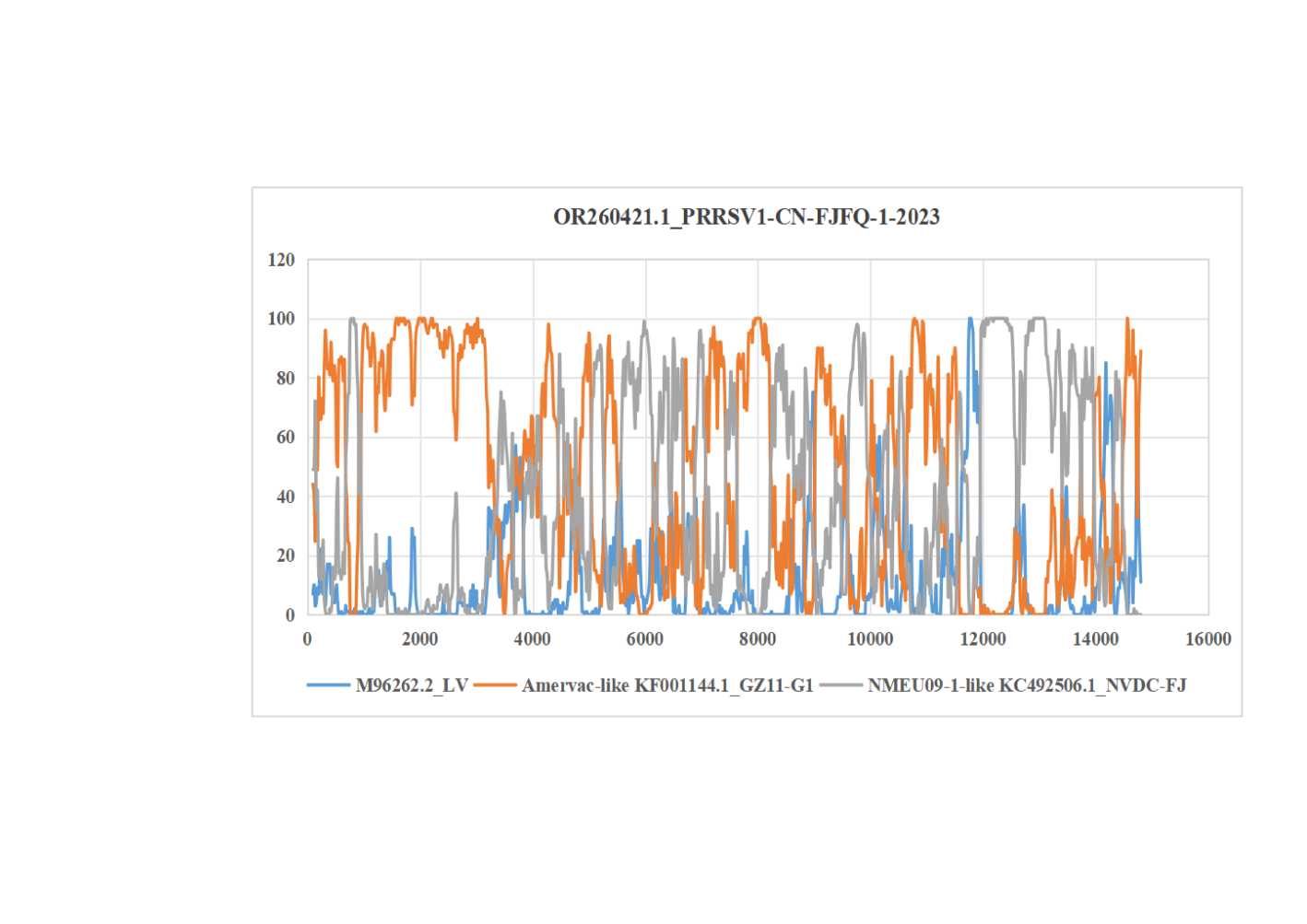


Supplementary Figure 1. Based on KT224385.1_HLJB1 and OR260421.1_PRRSV1-CN-FJFQ-1-2023 recombination event validation.(**A**):KT224385.1_HLJB1 Reconfiguration Event Validation.(**B**): OR260421.1_PRRSV1-CN-FJFQ-1-2023 Reconfiguration Event Validation.

Supplementary Table 2. Article 48 PRRSV whole genome sequence

| Sequence | Sequence |
| --- | --- |
| OQ606399.1_GD2022 | KT224385.1_HLJB1 |
| MK639926.1_EUGDHD2018 | KP860912.1_FJEU13 |
| OR260421.1_PRRSV1/CN/FJFQ-1/2023 | MN550991.1_KZ2018 |
| OR502390.1_PRRSV1/CN/FJFQ-4/2023 | KX967492.1_15HEN1_EU |
| EU076704.1_HKEU16 | MN927229.1_HL85 |
| KF287130.1_HK5 | OR333953.1_GXFS20220129 |
| KF287131.1_HK10 | PP330950.1_HLJTZJ155-2001 |
| KF287129.1_HK3 | OP566682.1_TZJ226 |
| KF287128.1_HK8 | OP566683.1_TZJ637 |
| GQ461593.1_SHE | OR636058.1_HBEU-328 |
| MN242825.1_NPUST-2789-3W-2 | PP330948.1_GDXNF94-1804 |
| KF001144.1_GZ11-G1 | PQ553567.1_PRRSV1/CN/FJEU02/2023 |
| M96262.2_LV | KP860913.1_FJQEU14 |
| GU047344.1_BJEU06-1 | GU047345.1_NMEU09-1 |
| MN927228.1_HeB47 | MK214314.1_P073-3 |
| JX187609.1_NVDC-NM1-2011 | KC492504.1_NVDC-NM2 |
| KM196101.1_LNEU12 | KC492505.1_NVDC-NM3 |
| MN927227.1_HeB3 | KC492506.1_NVDC-FJ |
| KY363382.1_HENZMD-10 | PP330949.1_GDXNF161-1806 |
| OP355712.1_ZD-1 | PQ640355.1_AHEU2024-2671 |
| MW115431.1_SC-2020-1 | OQ856755.1_PRRSV-1/181187-2/2023 |
| PP800763.1_SCPJ2023 | MK303390.1_180900-5 |
| PQ338766.1_GZ0308 | PP068352.1_AHB1 |
| OQ871594.1_SL-01 | AY150564.1_VR-2332 |

Note: The table contains 48 whole genome sequences, all of which are accession numbers_strain names.

Supplementary Figure 2. Recombination events involving the ORF1a-ORF7 genes in 32 recombinant events of the PRRSV-1 genome
